# A study on the clinical value of prophylactic contralateral central lymph node dissection in patients with cT1-T2N1b unilateral papillary thyroid cancer

**DOI:** 10.3389/fonc.2025.1629656

**Published:** 2025-07-16

**Authors:** Suqiong Lin, Rongliang Qiu, Yujuan Tang, Xiaoquan Hong, Qiangbin Ding, Ke Li, Ende Lin, Penghao Kuang, Jinbo Fu, Guoyang Wu

**Affiliations:** ^1^ Department of General Surgery, Zhongshan Hospital, Xiamen University, Xiamen, China; ^2^ The School of Clinical Medicine, Fujian Medical University, Fuzhou, China; ^3^ Department of Vascular Surgery, Hubei Provincial Hospital of Traditional Chinese Medicine, Wuhan, China; ^4^ Department of Ultrasound Medicine, Zhongshan Hospital, Xiamen University, Xiamen, China

**Keywords:** papillary thyroid carcinoma, lateral lymph node metastasis, contralateral central lymph node metastasis, preventive central lymph node dissection, surgical treatment

## Abstract

**Backgrounds:**

Lateral lymph node metastasis (cN1b) is a major factor affecting the prognosis and recurrence of papillary thyroid cancer (PTC). Currently, there is some controversy regarding whether to dissect the contralateral central lymph nodes in patients with cT1-T2N1b unilateral PTC. The purpose of this study was to investigate the risk factors for contralateral central lymph node metastasis (CCLNM) and to summarize the significance of prophylactic contralateral central lymph node dissection (CCLND), to provide reference information for clinical intervention.

**Methods:**

The data of 99 patients with cT1-T2N1b unilateral PTC from August 2021 to October 2024 were retrospectively analyzed. Multifactorial analysis was performed using logistic regression to analyze the risk factors for CCLNM in patients with cT1-T2N1b unilateral PTC. The analysis of the CCLNM rate and metastasis mode summarized the clinical significance of prophylactic CCLND.

**Results:**

CCLNM occurred in 55 cases (55/99,55.6%), and the total number of lymph nodes cleared from the contralateral central lymph node was 6.1 ± 4.9, of which the number of metastatic lymph nodes was 1.5 ± 1.9; There was no statistically significant difference between the CCLNM and non-metastasis groups in terms of the rate of lymph node metastasis in the ipsilateral lateral cervical region (zones II, III, IV and V) and the ipsilateral central zone (*P*>0.05). There was no statistically significant difference between the metastatic group and the non-metastatic group in terms of the number of lymph nodes cleared in the ipsilateral lateral cervical region (zones II, III, IV and V) (*P* > 0.05). Compared with the non-metastatic group, the metastatic group had more positive lymph nodes and fewer negative lymph nodes in the ipsilateral central region, and the difference was statistically significant (P < 0.05). Logistic regression analysis showed that microcalcification and Hashimoto’s thyroiditis in the metastasis group were independent factors for the occurrence of CCLNM, and the difference was statistically significant (*P*<0.05).

**Conclusion:**

The occurrence of CCLNM in cT1-T2N1b unilateral PTC is related to several factors. Lymph node dissection can help reduce the risk of recurrence and reoperation due to CCLNM; therefore CCLND cannot be ignored.

## 1 Introduction

In the United States, approximately 1.2% of people will be diagnosed with thyroid cancer at some point in their lives, with an estimated 44,020 new cases of thyroid cancer expected in 2025 (1). Over the past 40 years, with the widespread use of diagnostic imaging technology and the popularity of fine needle aspiration biopsy, the incidence of thyroid cancer has risen sharply, with an astonishing increase of 313% (2). Papillary thyroid carcinoma (PTC) is the most common type of thyroid cancer, accounting for approximately 84% of thyroid cancers (2). Although PTC progresses slowly and has a good overall prognosis, it is often accompanied by cervical lymph node metastasis in the early stages, and lymph node metastasis has been proven to be an important risk factor affecting the survival rate of PTC patients. At the same time, lymph node metastasis is closely related to a higher risk of local recurrence, distant metastasis and death (3).

The first stop for cervical lymph node metastases is usually the central lymph nodes, followed by the ipsilateral lateral cervical lymph nodes, then the contralateral cervical lymph nodes, and mediastinal lymph nodes. However, an increasing number of patients are clinically found to have direct involvement of lateral lymph node metastasis (LLNM) without central lymph node metastasis (CLNM), that is, ‘jumping lymph node metastasis’. The 2022 National Comprehensive Cancer Network (NCCN) guidelines and the 2015 American Thyroid Association (ATA) guidelines both support total thyroidectomy (TT) for patients with LLNM (zones IIzo) metastasis (N1b) (4, 5). For patients with cN1b unilateral PTC, TT + ipsilateral cervical lymph node dissection and ipsilateral central lymph node dissection are typically performed. However, a common problem encountered by thyroid surgeons in clinical practice is whether to perform prophylactic contralateral central lymph node dissection (CCLND) should be in patients with cT1-T2N1b unilateral PTC without malignant foci in the contralateral glandular lobe. In this regard, no uniform guidelines have been issued by domestic and international guidelines. However, according to domestic and international literature, the incidence of contralateral central lymph node metastasis (CCLNM) is approximately 20%-50% (6–10), and neglecting to clear the contralateral central lymph node greatly increases the probability of postoperative recurrence and distant metastasis and increases the likelihood of secondary surgery. Therefore, CCLND in the initial surgery can reduce the probability of lymph node recurrence, make the TNM staging more accurate, and help the choice of subsequent treatment options. However, at the same time, this also increases the difficulty of surgery and the probability of postoperative complications for surgeons to a certain extent. This study aimed to further analyze the clinical significance of preventive CCLND in patients with cT1-T2N1b unilateral PTC, explore high-risk factors for CCLNM, and provide reference materials for clinical intervention.

## 2 Material and methods

### 2.1 General information

**Figure 1:**
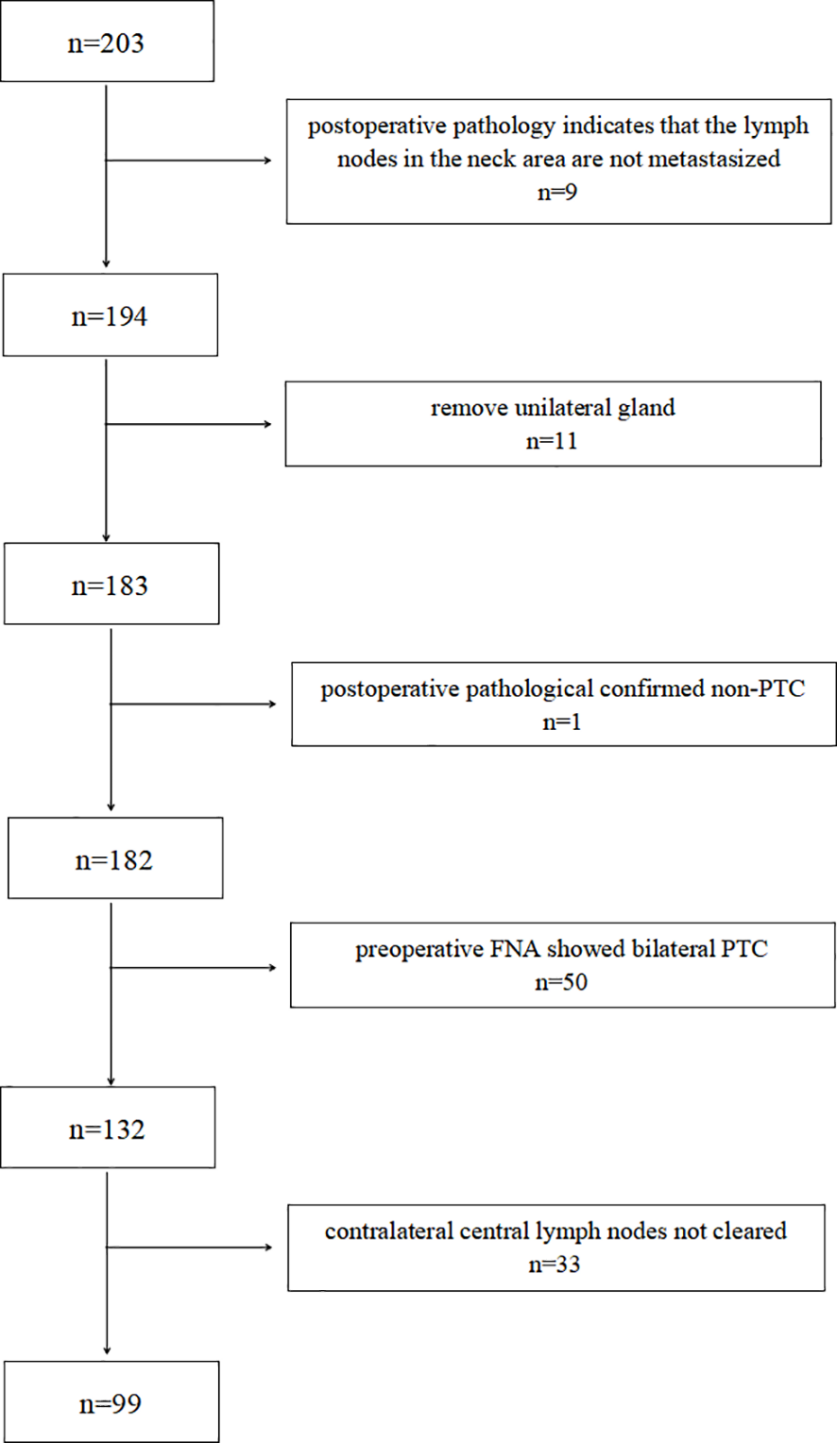
Patient screening flowchart.

A retrospective analysis was performed on 400 N1b PTC patients admitted to the Department of General Surgery of Zhongshan Hospital, Xiamen University, from August 2021 to October 2024. Based on the inclusion and exclusion criteria, 99 patients with cT1-T2N1b unilateral PTC were included in the study. A patient screening flowchart is presented in **Figure 1**.

The inclusion criteria: ①unilateral PTC with LLNM confirmed by postoperative pathology; ②TT and ipsilateral cervical lymph node dissection combined with bilateral central lymph node dissection; ③lesions were located in unilateral lobes only; ④patients were examined by imaging examination and ultrasound-guided fine-needle puncture of cervical lymph nodes prior to the operation, which fulfilled the criteria of cervical lymph node positivity; ⑤ Preoperative imaging studies did not reveal any suspicious metastatic lymph nodes in the contralateral central lymph node region. ⑥complete clinical and pathological data. The exclusion criteria. ①history of previous neck surgery or radiotherapy; ②postoperative pathology of non-PTC; ③history of other malignant tumors causing cervical lymph node metastasis; ④those severe organic diseases that cannot tolerate surgery.

This study was approved by the Medical Ethics Committee of Zhongshan Hospital, Xiamen University, china. The study design and writing followed the Strengthening the Reporting of Observational Studies in Epidemiology (STROBE) guidelines.

### 2.2 Surgical procedures

A tracheal tube with neuromonitoring was placed through the mouth and general anesthesia was administered. The patient was placed in a natural supine position, with a filler placed under the shoulder and the neck slightly stretched. All the patients underwent total thyroidectomy and ipsilateral cervical lymph node dissection combined with bilateral central lymph node dissection. The subcutaneous operating space was established according to the surgical approach selected by the patient prior to surgery. The white line of the neck was incised to expose the thyroid gland and the thyroid glands on the affected and contralateral sides were removed. During the fine dissection during the operation, attention was paid to protecting important tissues, such as the parathyroid gland and the recurrent laryngeal nerve. The ipsilateral cervical lymph nodes (zones IIon), the ipsilateral central lymph nodes (zones VI), and the contralateral central lymph nodes (zones VI) were dissected in turn. The scope of cervical lymph node dissection was along the medial edge of the sternocleidomastoid muscle, dissected upward to the digastric plane; along the internal jugular vein, dissected to the vagus nerve; along the subclavian free, dissected downward to the subclavian vein, and continued to be freed outward to the anterior edge of the trapezius muscle. The scope of lymph node dissection in the central neck area was upward to the hyoid plane, outward to the lateral edge of the common carotid artery on both sides, and downward to the level of the innominate artery. All lymph nodes and fat tissues in front of the trachea, in front of the larynx, and beside the tracheoesophagus, that is, above the innominate artery behind the manubrium of the sternum, are cleared. When removing the lower pole and pretracheal lymph nodes, the lymph nodes and fat tissues were pulled upward to the thymus. When cleaning, the differences in anatomical structures on the left and right sides should be noted to reduce the possibility of omission. Finally, the white line of the neck was sutured and a drainage tube was placed.

### 2.3 Observation indicators

All patients had preoperative primary thyroid lesions and suspicious cervical lymph nodes, as confirmed by ultrasound-guided thyroid fine-needle aspiration pathology. Preoperative neck or thyroid ultrasonography, enhanced CT, laryngoscopy, and other relevant examinations were performed. Postoperative pathological data were recorded, including extraglandular infiltration, ipsilateral LLNM metastasis, ipsilateral CLNM metastasis, and CCLNM.

The patients were discharged from the hospital without any obvious discomfort, in good general condition, with drains removed, and wounds healing well without obvious redness, swelling, hardness, hematoma, or effusion. All patients received thyroid-stimulating hormone suppression therapy postoperatively. According to the guidelines and based on the postoperative pathological staging, it is recommended that patients undergo radioactive iodine therapy in the oncology department 1–3 months after surgery.

### 2.4 Statistics

SPSS software (version 26.0) was used to process data. Continuous variable data are expressed as mean ± standard deviation (x ± s), and the independent sample t-test was used. Categorical variable data are expressed as n (%) and the X^2^ test was used. Logistic regression analysis was used to analyze the risk factors of CCLNM in patients with unilateral PTC and LLNM. Statistical significance was set at *P* < 0.05.

## 3 Results

### 3.1 Comparison of CCLNM and clinical related factors

As shown in **Table 1**, the proportion of microcalcification and Hashimoto thyroiditis (HT) in the CCLNM group was significantly lower than that in the non-metastatic group (*P*<0.05).

**Table 1:** Analysis of the correlation between contralateral central lymph node metastasis and clinical factors.

Clinically relevant factors	Category	N	CCLNs		*t*/*Z*/*χ²*	*P* value
			Yes (n=55)	No (n=44)		
Age (years)		99	42.9 ± 13.5	41.4 ± 13.5	-0.634	0.528
Gender	Female	63	32 (58.2)	31 (70.5)	1.591	0.207
	Male	36	23 (41.8)	13 (29.5)		
BMI (kg/m^2^)			22.8 ± 2.6	22.8 ± 2.9	0.030	0.976
Tumor diameter (cm)			1.6 (1.1-2.1)	1.5 (1.0-2.0)	-0.811	0.417
Tumor number	Unifocal	69	37 (67.3)	32 (72.7)	0.344	0.557
	Multifocal	30	18 (32.7)	12 (27.3)		
Tumor location	Upper Pole	36	20 (36.4)	16 (36.4)	3.353	0.187
	Middle Pole	37	17 (30.9)	20 (45.5)		
	Lower Pole	26	18 (32.7)	8 (18.2)		
extraglandular infiltration	Yes	66	34 (61.8)	32 (72.7)	1.309	0.253
Microcalcification	Yes	30	12 (21.8)	18 (40.9)	4.218	0.040
Hashimoto thyroiditis	Yes	39	15 (27.3)	24 (54.5)	7.615	0.006

CCLNs, Contralateral central lymph node metastases.

### 3.2 Comparison in terms of lymph node metastasis rate and number of cleared lymph nodes

CCLNM occurred in 55 cases (55/99,55.6%), and the total number of lymph nodes cleared from the contralateral central lymph node was 6.1 ± 4.9, of which the number of non-metastatic lymph nodes was 4.7 ± 4.6, and the number of metastatic lymph nodes was 1.5 ± 1.9; ipsilateral CLNM occurred in 91 cases (91/99,91.9%), bilateral CLNM occurred in 53 cases (53/99,53.5%), and ipsilateral CLNM without CCLNM occurred in 38 cases (38/99,38.4%).

There was no statistically significant difference between the CCLNM and the non-metastasis groups in terms of the rates of ipsilateral lateral cervical zone (zones II, III, IV and V) and ipsilateral CLNM (*P*>0.05), as shown in **Table 2**.

**Table 2:** Analysis of the correlation between lymph node metastasis in different zones and contralateral central lymph node metastasis.

Lymph nodes in different divisions	Positive lymph node	N	CCLNs		*χ²*	*P* value
			Yes (n=55)	No (n=44)		
Ipsilateral lateral cervical lymph node	Yes	99	55 (100.0)	44 (100.0)		
Zone II lymph nodes	Yes	50	31 (56.4)	19 (43.2)	1.699	0.192
Zone III lymph nodes	Yes	76	44 (80.0)	32 (72.7)	0.725	0.395
Zone IV lymph nodes	Yes	76	41 (74.5)	35 (79.5)	0.343	0.558
Zone V lymph nodes	Yes	4	1 (1.8)	3 (6.8)	0.550	0.458
Ipsilateral central lymph node	Yes	91	53 (96.4)	38 (86.4)	2.082	0.149

Comparing the metastasis group with the non-metastasis group in terms of the total number of lymph nodes, the total number of positive lymph nodes, and the total number of negative lymph nodes in the ipsilateral cervical zone clearance (zones II, III, IV and V), the difference was not statistically significant (*P*>0.05); the metastasis group, compared with the non-metastasis group, had more total positive and fewer total negative lymph nodes in the ipsilateral central zone clearance, and the difference was statistically significant (*P*<0.05). For more details, see **Table 3**.

**Table 3:** Number of lymph nodes cleared.

Lymph nodes in different divisions	LN category	CCLNs		*t*/*Z*	*P* value
		Yes (n=55)	No (n=44)		
Ipsilateral lateral cervical lymph node	Total Retrieved LN	32.5 ± 11.3	35.0 ± 14.1	0.977	0.331
	Positive LN	5.0 (3.0-7.0)	5.0 (2.0-6.5)	-0.121	0.904
	Negative LN	27.3 ± 10.9	29.7 ± 13.4	0.972	0.333
Zone II lymph nodes	Total Retrieved LN	10.0 (6.0-13.0)	9.0 (6.5-11.5)	-0.420	0.674
	Positive LN	1.0 (0.0-1.0)	0.0 (0.0-1.5)	-0.882	0.378
	Negative LN	9.0 (5.0-12.5)	8.0 (6.0-10.5)	-0.237	0.813
Zone III lymph nodes	Total Retrieved LN	9.0 (7.0-12.0)	9.0 (5.5-14.0)	-0.028	0.977
	Positive LN	2.0 (1.0-3.0)	1.0 (0.0-3.0)	-1.354	0.176
	Negative LN	7.0 (5.0-10.0)	7.0 (4.0-11.5)	-0.293	0.770
Zone IV lymph nodes	Total Retrieved LN	10.0 (6.0-14.0)	11.0 (7.5-17.5)	-1.097	0.273
	Positive LN	2.0 (0.5-3.0)	2.0 (1.0-4.0)	-0.673	0.501
	Negative LN	8.0 (5.0-11.0)	9.0 (4.0-15.0)	-0.808	0.419
Zone V lymph nodes	Total Retrieved LN	0.0 (0.0-2.0)	0.0 (0.0-2.0)	-0.188	0.850
	Positive LN	0.0 (0.0-0.0)	0.0 (0.0-0.0)	-1.269	0.204
	Negative LN	0.0 (0.0-2.0)	0.0 (0.0-1.5)	-0.017	0.986
Ipsilateral central lymph node	Total Retrieved LN	7.0 (4.0-10.5)	8.5 (5.0-12.0)	-1.003	0.316
	Positive LN	4.0 (2.0-5.5)	3.0 (1.0-5.0)	-1.984	0.047
	Negative LN	3.0 (1.0-5.5)	4.0 (2.0-7.5)	-2.298	0.022

LN, lymph node.

### 3.3 Logistic regression analysis of cT1-T2N1b unilateral PTC patients who developed contralateral central regional lymph node metastasis

The statistically significant indicators in **Tables 1**, **2** were included in the logistic multifactorial regression analysis. The results showed that microcalcification and HT were independent factors for the occurrence of CCLNM, and the difference was statistically significant (P<0.05) (**Table 4**).

**Table 4:** Logistic regression analysis of contralateral central lymph node metastases.

Variable	*B*	*SE*	*Wald X^2^*	*P* value	*OR*	95% *CI*
Microcalcification	-1.123	0.481	5.463	0.019	0.325	0.127-0.834
Hashimoto thyroiditis	-1.326	0.452	8.606	0.003	0.265	0.109-0.644

## 4 Discussion

Lymph node metastasis in the lateral cervical region is a major factor affecting the prognosis and recurrence of PTC as well as a factor that determines the extent of resection of the primary lesion. In the past, when performing thyroid cancer resection, selective lymph node dissection was usually adopted to reduce the occurrence of secondary surgery. According to the ATA and NCCN guidelines, prophylactic central lymph node dissection should be considered for PTC patients with advanced tumor stage cN0-1b (4, 5). However, it has also been suggested that prophylactic central zone lymph node dissection is not necessary if the tumor is small, no peritumoral invasion is seen and the central lymph nodes are negative during preoperative imaging assessment (11). Immediate ultrasound is an accurate tool for identifying metastatic lymph nodes (12), but suspicious ultrasound signs (round, cystic changes, hyperechoic lesions or microcalcifications, irregular chaotic vascular formations, and loss of lymphatic gates) are not highly specific (13). At the same time, the diagnostic performance of computed tomography scans is influenced by the subjective judgment of the radiologist, which leads to inaccurate staging assessment or understaging or over-staging in a significant proportion of patients. Studies have shown that occult metastases can be detected in the central region in 40%-70% of cases, and because of the high rate of occult metastases and even clinicopathological findings of micrometastases in about 90% of patients, prophylactic central lymph node dissection can help detect them, and consequently, to further change the staging of PTC and the adjuvant therapeutic regimen in the postoperative period (14, 15). The present study focused on patients with cT1-T2N1b unilateral PTC, aiming to investigate the feasibility of contralateral central lymph node dissection and to analyze the high-risk factors for CCLNM in this group of patients, with the aim of providing a strong basis for controlling the recurrence rate of the disease and improving the prognosis.

The central regions of the neck (pre-laryngeal, pre-tracheal, and paratracheal tissues) have a high number of interconnected branches through which tumor cells can metastasize from one central region to the contralateral central region, thus causing lymph node metastasis in the contralateral central region. Although PTC cell multiplication occurs in a predictable stepwise manner through the orderly arranged lymphatic system, jump metastases can also occur when uninvolved contiguous areas are interspersed with involved tumors (16). The possibility of involvement of the contralateral central lymph nodes in patients with jump metastases in the unilateral lateral cervical zone has not been fully clarified. In this study, we conducted an in-depth analysis of the clinical factors affecting the occurrence of metastases in the contralateral central zone, and the results showed a correlation between CCLNM and microcalcifications and HT. Although previous studies have explored the risk factors for cervical lymph node metastasis in patients with PTC, the findings remain controversial. The presence of microcalcifications on ultrasound is usually indicative of a significant proliferation of fibrous tissue and tumor vasculature within the lesion, accompanied by calcification and deposition of cancer cells, which is a specific indicator for the diagnosis of PTC and an important factor contributing to the increased risk of metastasis (17, 18). Previous studies have also confirmed a correlation between CLNM and microcalcifications, with microcalcifications increasing the risk of CLNM. This finding is inconsistent with the results of this study. However, the primary reason for this discrepancy is the relatively small sample size included in this study. Additionally, the cases included in this study were limited to patients with cT1-T2N1b unilateral PTC. These factors remind us that when exploring the impact of microcalcification differences, it is essential to carefully consider the limitations that may exist in research methods and data analysis, while also being mindful of potential biases in patient selection. Future studies should further expand the sample size and conduct a more in-depth exploration of the relationship between cN0 PTC and microcalcifications. A retrospective study in Korea that in patients with PTC accompanied by HT, although the number of lymph nodes cleared by central lymph node dissection was increased, it did not increase the detection rate of positive lymph nodes, and at the same time, it showed that HT was independently correlated with good prognosis in patients with PTC (19). This finding is consistent with the results of the present study and supported by other studies (20, 21). This may be due to the characteristic high concomitant nature of HT in PTC as well as the fact that HT causes cervical lymph node enlargement through a chronic inflammatory immune response, resulting in reactive lymph node hyperplasia frequently observed in the central region (19, 22). In actual surgery, the number of central lymph node dissections inevitably increases and the percentage of positive lymph nodes is somewhat reduced.

In addition, the prevailing view is that patients with PTC with larger tumor size, located at the upper pole, and multifocal PTC have more aggressive tumors, which may also be risk factors for the occurrence of lymph node metastasis (20, 23, 24). However, this conclusion was not reached in this study. Tumor diameter is critical for postoperative TNM pathological staging and prognosis risk assessment in PTC patients. It is positively correlated with growth rate and tissue invasion extent and is more likely to result in central lymph node metastasis (25). The ATA guidelines recommend routine prophylactic central lymph node dissection for patients with T3 or T4 tumors, as the risk of lymph node metastasis increases in patients with tumor diameters of 4 cm or larger (5). Based on the Surveillance, Epidemiology, and End Results (SEER) database, a multivariable analysis was conducted on thyroid cancer patients who underwent surgical treatment between 2002 and 2012 (n=80,565), and the results confirmed a correlation between tumor diameter ≥i cm and central region metastasis (25). This study showed no statistically significant association between tumor diameter and CCLNM. The primary reasons for this outcome include: first, cases were strictly limited to T1–2 stages (tumor diameter ≤4 cm), and a stratified risk analysis was conducted based on tumor diameter; Second, sample size limitations. These findings suggest that tumor diameter may not be a necessary indicator for CCLND in the management of early-stage PTC (T1–2 stages).

In this study, the ipsilateral CLNM rate was as high as 91.9% (91/99), CCLNM rate was 55.6% (55/99), and bilateral CLNM rate was 53.5% (53/99). Among the patients without ipsilateral CLNM, 25.0% (2/8) had positive contralateral central lymph nodes. Some studies have shown that the high or low rate of cervical lymph node metastasis in patients with PTC is closely related to their specific metastasis pattern, and this metastasis follows a certain zoning rule (26). Lymph node metastasis usually first occurs in the central lymph nodes (anterior laryngeal, anterior tracheal, and paratracheal tissues), and lymphatic vessels communicate with each other between the bilateral central zones. Metastasis of the central lymph nodes on one side can cause tumor cells to metastasize to the contralateral central zone through lymphatic vessel branches (16). In this study, the lymph node metastasis rates of patients in the cervical zones II, III, IV and V were 50.5% (50/99), 76.8% (76/99), 76.8% (76/99), and 5.1% (5/99), respectively, with the main metastasis occurring in zones III and IV. Therefore, from the perspective of the lateral cervical lymph node metastasis rate or the overall law of cervical lymph node metastasis, when lateral cervical lymph node metastasis occurs, the risk of metastasis in the contralateral central zone cannot be ignored. Therefore, it is necessary to perform preventive CCLND in patients with PTC in clinical practice. The ATA and NCCN guidelines also believe that patients with clinically positive lymph nodes (cN1) are suitable candidates for preventive central lymph node dissection (4, 5). However, the main argument against prophylactic central lymph node dissection is its possible increased risk of surgical complications (27, 28). Among these complications, parathyroid injury and recurrent laryngeal nerve injury are the most common and may result in temporary or permanent hypocalcaemia and hoarseness. The incidence of temporary and permanent hypocalcaemia has been reported to be as high as 44% and 4%, respectively, and temporary and permanent recurrent laryngeal nerve injuries are as high as 7.3% and 3.6%, respectively (29). However, Chae et al. found that unilateral versus bilateral central lymph node dissection resulted in no significant difference in the incidence of laryngeal recurrent nerve injury and temporary hypocalcaemia (8). The results of a meta-analysis also showed that total thyroidectomy alone and total thyroidectomy combined with prophylactic central lymph node dissection did not differ in the incidence of permanent hypoparathyroidism and temporary or permanent recurrent laryngeal nerve injury (30). Other researchers have reached similar conclusions and found no statistically significant difference between the incidence of permanent hypoparathyroidism and permanent recurrent laryngeal nerve injury in these two groups (31–33). Therefore, it is recommended that the operation be performed by an experienced surgeon familiar with surgical anatomy, with precise preoperative localization using tracers and intraoperative dissection using advanced stripping equipment to preserve the parathyroid glands and the recurrent laryngeal nerve, thus effectively avoiding postoperative complications. If prophylactic dissection can be performed at the time of the initial surgery, it can avoid the short-term metastatic spread of cancer cells due to missed lymph node dissection, thus reducing the probability of metastasis and recurrence (31, 32, 34). In addition, this can avoid the possibility of having to undergo a secondary operation because of the occurrence of metastasis and recurrence. Secondary surgery will not only increase the overall difficulty of surgery, such as scar adhesion and anatomical structure changes, but also increase the incidence of surgical complications, which will cause great physical and psychological burden to the patients (32, 35). In summary, prophylactic clearance of contralateral central lymph nodes not only helps to remove undetected involved lymph nodes and reduces the probability of postoperative recurrence and metastasis but also more accurately determines the pathological stage of postoperative TNM, which provides more accurate information for patients’ postoperative treatment options, follow-up protocols, and assessment of the risk of postoperative recurrence, and thus greatly improves the patient’s prognosis.

The limitations of this study are as follows: Firstly, as a retrospective study, its inherent design makes it difficult to completely avoid the problem of potential bias. Second, this study was a single-center study with a relatively small sample size, which limited our ability to comprehensively assess the impact of prophylactic central lymph node dissection on patients’ long-term prognosis and quality of life. To overcome these limitations and obtain more accurate and comprehensive findings, we plan to conduct multicenter, large-sample, randomized controlled clinical studies in the future, with a view to providing more reliable and robust evidence support in this area.

## 5 Conclusion

Based on the above analysis of the metastasis rate, metastasis pattern, and clinical risk factors of the contralateral central lymph nodes in patients with cT1-T2N1b unilateral PTC, this study suggests that the CCLND should not be ignored in patients with unilateral cN1b. Metastasis of the contralateral central lymph nodes in cT1-T2N1b unilateral PTC is closely association with many factors. CCLND can help reduce the risk of recurrence and reoperation due to contralateral lymph node metastasis, thereby providing patients with a more comprehensive and effective treatment strategy.

## Data Availability

The original contributions presented in the study are included in the article/supplementary material. Further inquiries can be directed to the corresponding authors.
